# Polyphasic taxonomy of glacier-derived Arthrobacter strains reveals six novel species within the Arthrobacter agilis group and indicates Arthrobacter vasquezii Valenzuela-Ibaceta et al. 2023 as a later heterotypic synonym of Arthrobacter parietis Heyrman et al. 2005

**DOI:** 10.1099/ijsem.0.007246

**Published:** 2026-07-21

**Authors:** Lei-Lei Yang, Yu-Hua Xin, Qing Liu

**Affiliations:** 1 State Key Laboratory of Microbial Diversity and Innovative Utilization, Institute of Microbiology, Chinese Academy of Sciences, Beijing 100101, PR China; 2 China General Microbiological Culture Collection Center (CGMCC), Institute of Microbiology, Chinese Academy of Sciences, Beijing 100101, PR China; 3 Beijing Key Laboratory of Genetic Element Biosourcing & Intelligent Design for Biomanufacturing, Beijing 100101, PR China

**Keywords:** *Arthrobacter*, *Arthrobacter vasquezii*, *Arthrobacter parietis*, glacier, later heterotypic synonym

## Abstract

Eight bacterial strains assigned to the genus *Arthrobacter* by preliminary 16S rRNA gene screening were isolated from supraglacial samples collected from four glaciers in China. Genome-based analyses provided the primary evidence for species delineation. Pairwise average nucleotide identity (ANI) and digital DNA–DNA hybridization (dDDH) comparisons among the eight strains and between the glacier-derived strains and their closest type-strain relatives supported their assignment to six novel species, with strains HZ1^T^ and HLT1-21 (ANI 99.1%, dDDH 92.2%) and strains MDB2-24^T^ and MDT3-44 (ANI 98.5%, dDDH 87.9%) each representing a single genomic species. Consistently, a maximum-likelihood phylogenomic tree inferred from 81 concatenated bacterial core genes resolved 6 well-supported independent species-level lineages within the *Arthrobacter agilis* group. The predominant cellular fatty acid of the representative strains was anteiso-C_15 : 0_ (34.5–66.1%). In addition, genome-relatedness indices indicated that *Arthrobacter vasquezii* Valenzuela-Ibaceta *et al.* 2023 is conspecific with *Arthrobacter parietis* Heyrman *et al.* 2005 (ANI 98.8%, dDDH 90.1%) and should, therefore, be treated as a later heterotypic synonym of *A. parietis* Heyrman *et al.* 2005 under the principle of priority. Based primarily on phylogenomics and genome relatedness, supplemented by phenotypic and chemotaxonomic characterization, the eight strains represent six novel species of the genus *Arthrobacter*, for which the following names are proposed: *Arthrobacter glycogeni* sp. nov. (HZ1^T^=CGMCC 1.9261^T^=JCM 38152^T^), *Arthrobacter arabinosi* sp. nov. (TMS1-12-1^T^=CGMCC 1.9392^T^=NBRC 113087^T^), *Arthrobacter melezitosi* sp. nov. (TMN-37^T^=CGMCC 1.9692^T^=JCM 38156^T^), *Arthrobacter sorbitolis* sp. nov. (MDT1-65^T^=CGMCC 1.9836^T^=JCM 38163^T^), *Arthrobacter rhamnosi* sp. nov. (MDB2-24^T^=CGMCC 1.9862^T^=JCM 38160^T^) and *Arthrobacter pasteuri* sp. nov. (MDT2-2^T^=CGMCC 1.9874^T^=JCM 38164^T^).

The genus *Arthrobacter*, established by Conn and Dimmick [1], belongs to the family *Micrococcaceae* and comprises aerobic, Gram-stain-positive actinobacteria that typically display a rod-coccus life cycle and are widely distributed across terrestrial and aquatic environments [2]. Taxonomic boundaries within *Arthrobacter sensu lato* have been extensively revised in response to phylogenetic and chemotaxonomic heterogeneity, leading to the emendation of *Arthrobacter* and the establishment of several related genera (e.g. *Glutamicibacter*, *Paenarthrobacter* and *Pseudarthrobacter*) [2]. Despite these revisions, species-level circumscription within *Arthrobacter* remains challenging because several validly published species share >98% 16S rRNA gene sequence similarity, and population genetic analyses have shown evidence for extensive homologous recombination within *Arthrobacter sensu lato* [3]. Consequently, 16S rRNA gene data are useful for preliminary genus-level assignment and for interpreting amplicon-based surveys, but genome-scale comparisons are required for robust species delimitation in closely related *Arthrobacter* species.

Glacier surfaces and cryoconite granules represent cold, oligotrophic and spatially heterogeneous microhabitats where actinobacteria are recurrent components of the microbial community [4,5]. Cultivation-based surveys have repeatedly recovered *Arthrobacter* from cryoconite and glacier-associated samples, supporting the view that members of this genus contribute to heterotrophic carbon turnover and persistence under cold stress [6,7]. Such spatially structured microhabitats may favour the coexistence of closely related but genomically distinct species. Nevertheless, the present sampling is insufficient to determine whether these species are endemic or broadly distributed; these biogeographic questions will require expanded genome sampling from additional glacier and non-glacier habitats. In the present study, eight *Arthrobacter* strains were isolated from glacier-surface samples collected across geographically separated glaciers in China. Polyphasic characterization, integrating phylogenomic inference and comparative phenotypic analyses, demonstrated that these isolates represent six novel species within the *Arthrobacter agilis* group [2].

## Isolation and ecology

Eight bacterial strains (HZ1^T^, TMS1-12-1^T^, TMN-37^T^, MDT1-65^T^, MDB2-24^T^, MDT2-2^T^, MDT3-44 and HLT1-21) were isolated from supraglacial samples collected from glaciers located across northwestern and southwestern China (Table S1). Sampling sites included Xinjiang No. 1 Glacier (Xinjiang, China), Toumingmengke Glacier (Gansu, China), Midui Glacier (Xizang, China) and Hailuogou Glacier (Sichuan, China) and the isolates originated from glacier-surface materials (e.g. cryoconite/ice-associated samples) as recorded in Table S1 (available in the online Supplementary Material). Samples were aseptically collected, homogenized and serially diluted in sterile water. Aliquots were spread onto peptone-yeast extract-glucose (PYG) agar (0.5% Bacto peptone, 0.02% yeast extract, 0.5% glucose, 0.3% beef extract, 0.05% NaCl and 0.15% MgSO_4_·7H_2_O; pH 7.0). Plates were incubated at 14 °C for up to 15 days. This temperature was selected as a low-temperature cultivation condition suitable for recovery of psychrotolerant glacier bacteria while still allowing colony development within a practical incubation period. Colonies were purified by repeated streaking on PYG agar. Unless otherwise stated, strains were routinely maintained on PYG agar at 14 °C. Long-term preservation was performed in 10% (v/v) glycerol in liquid nitrogen.

## 16S rRNA phylogeny

Genomic DNA of the eight strains was extracted using the TaKaRa MiniBEST Bacteria Genomic DNA Extraction Kit v3.0 (TaKaRa, Japan) following the manufacturer’s instructions. Nearly complete 16S rRNA gene sequences were amplified by PCR with the universal primer pair 27F/1492R [8] and determined by Sanger sequencing. Closest phylogenetic neighbours were identified by querying the resulting sequences against the EzBioCloud reference database [9]. Multiple sequence alignments were generated with MAFFT v7.5 [10]. Neighbour-joining (NJ) and maximum-likelihood (ML) phylogenetic trees were reconstructed in mega v12 [11]. For the NJ tree, evolutionary distances were computed under the Kimura two-parameter model, and nodal robustness was evaluated using 1,000 bootstrap replicates. For the ML analysis, the best-fitting nucleotide substitution model TN93+G+I was selected in mega v12, and nodal support was evaluated using 1,000 bootstrap replicates. The 16S rRNA gene analyses were used only for preliminary genus-level placement and for selecting strains requiring genome-based taxonomic characterization.

Nearly full-length 16S rRNA gene sequences were obtained by Sanger sequencing and additionally retrieved from the assembled genomes. EzBioCloud and blast comparisons assigned all eight isolates to the genus *Arthrobacter*. The isolates showed high sequence similarity to multiple described *Arthrobacter* species, including several similarities above 98%, confirming that this marker has limited discriminatory power for species-level circumscription in this group.

The NJ and ML analyses of 16S rRNA gene sequences gave congruent genus-level placement within *Arthrobacter* (Figs S1 and S2), but several internal relationships were weakly supported or unresolved. In particular, the strain pairs HZ1^T^/HLT1-21 and MDB2-24^T^/MDT3-44 were not discriminated by 16S rRNA gene phylogeny. We, therefore, did not use the 16S rRNA gene tree as species-delimitation evidence; instead, species boundaries were evaluated primarily using phylogenomics, average nucleotide identity (ANI) and digital DNA–DNA hybridization (dDDH).

## Genome features

For whole-genome sequencing, paired-end libraries (150 bp) were prepared according to standard Illumina protocols and sequenced on a HiSeq 4000 platform (Illumina, San Diego, CA, USA). After quality filtering, reads were assembled *de novo* with SPAdes v3.15 [12]. Assembly metrics were summarized with QUAST v5.0.2 [13], and genome completeness/contamination were estimated using CheckM2 v1.0.2 [14]. Automated genome annotation was performed with Prokka v1.14 [15]. Reference taxa for phylogenomic and genome-relatedness analyses were selected as type-strain genomes of validly published species in the *A. agilis* group and closely related *Arthrobacter* taxa identified by EzBioCloud/blast searches. Genomes were retrieved from NCBI RefSeq/GenBank; the full list of reference genomes and accession numbers is provided in Table S2. For phylogenomic reconstruction, 81 bacterial single-copy core genes were identified using UBCG2 (default parameters) [16]. The concatenated nucleotide alignment was generated with MAFFT v7.5 [10], and poorly aligned regions were removed using Gblocks v0.91b before tree construction [17]. Model selection and maximum-likelihood tree inference were conducted in IQ-TREE 2 [18]; the final tree was inferred using the GTR+F+R7 substitution model with branch support assessed by 1,000 bootstrap replicates. Pairwise ANI values were calculated using FastANI v1.33 [19] between the eight glacier-derived strains and their relatives listed in Table S2. Based on the ANI results and phylogenomic relationships, the closest type-strain relatives of the proposed novel species were selected for further dDDH analysis. dDDH values were estimated using the Type (Strain) Genome Server with the recommended formula *d4* [20]. Thus, ANI was used for comprehensive genome screening, whereas dDDH was used as an independent confirmatory measure for selected closest genome pairs.

Draft genomes were obtained for all eight strains using Illumina paired-end sequencing followed by *de novo* assembly and standardized quality assessment. Assemblies exhibited high estimated completeness (99.91–100%) and low contamination (0.02–0.42%) (Table S3). Genome sizes ranged from 3.44 to 3.80 Mb, with DNA G+C contents of 63.7–69.6 mol%. Assemblies comprised 10–53 contigs and showed N50 values spanning 135,230–2,317,634 bp, consistent with high-quality draft genomes appropriate for genome-based taxonomy. Automated genome annotation predicted 3,190–3,508 protein-coding sequences and 3,260–3,583 total genes per genome. Non-coding features comprised 14–28 miscellaneous RNAs, 3–5 rRNA genes, 48–52 transfer RNAs and one transfer–messenger RNA in each assembly (Table S4).

**Fig. 1.**
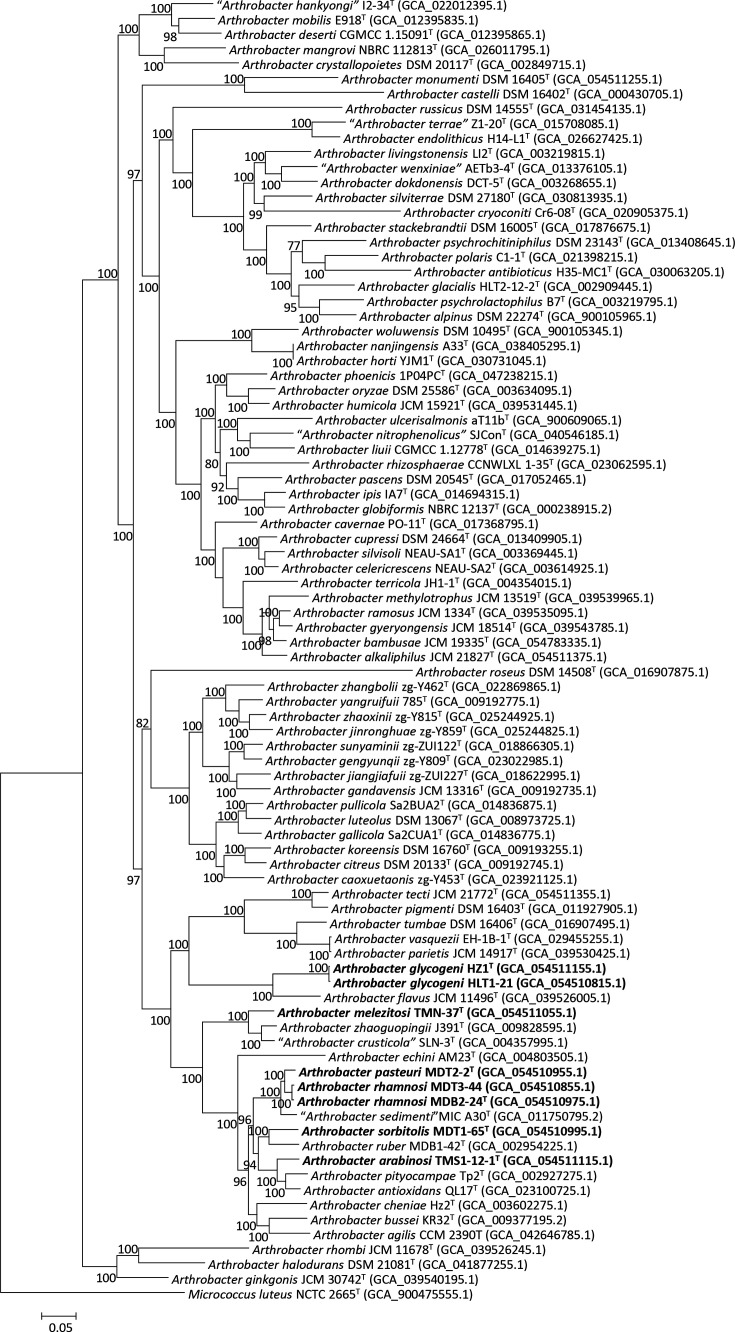
Phylogenetic tree of the eight strains and related taxa, including *Arthrobacter vasquezii* EH-1B-1^T^ and *Arthrobacter parietis* JCM 14917^T^, inferred using the ML method with the GTR+F+R7 model based on a concatenated nucleotide alignment of 81 bacterial core genes. Bootstrap values (>50%) from 1,000 replicates are shown at branch nodes. Scale bar, 0.05 substitutions per nucleotide position.

**Fig. 2.**
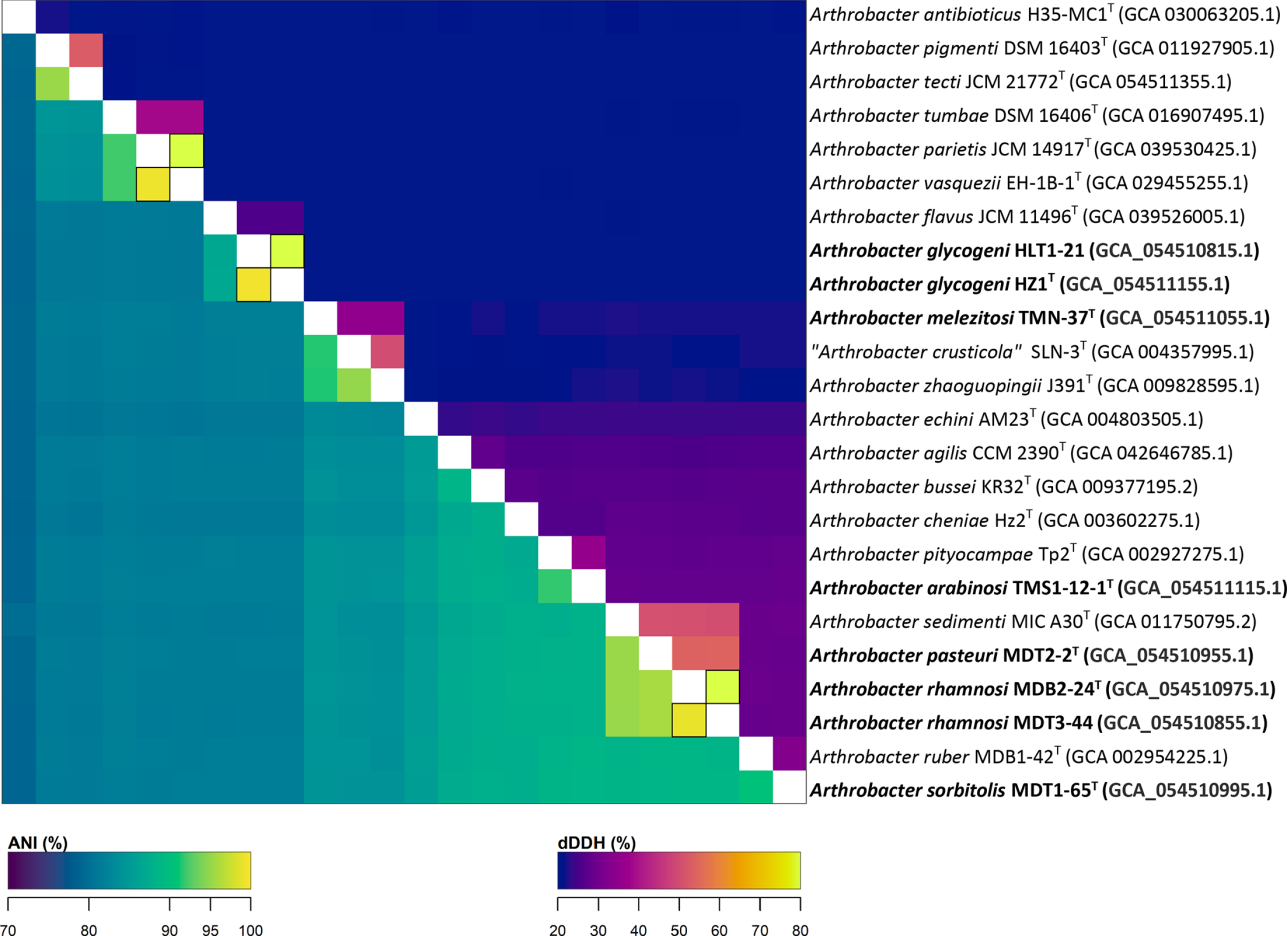
Heatmap of pairwise genomic relatedness among the eight glacier-derived strains and selected closely related type strains, with ANI values (%) shown in the lower triangle and dDDH values (%) in the upper triangle. Genome pairs exceeding both thresholds applied in this study – ANI>96% and dDDH>70% – are highlighted with black outlines, indicating conspecific-level genomic relatedness.

Phylogenomic inference based on a concatenated nucleotide alignment of 81 bacterial core genes (UBCG2) resolved the eight glacier-derived strains within the *Arthrobacter agilis* group [2] and separated them into six well-supported, independent species-level lineages (Fig. 1). ANI values were calculated between the eight glacier-derived strains and all reference genomes listed in Table S2. Based on these results, the closest type-strain relatives of the proposed novel species were selected for further dDDH analysis. The ANI and dDDH values for the genome pairs included in the comparative heatmap are shown in Fig. 2. Among the eight glacier-derived strains, two strain pairs exceeded the applied species-level thresholds (ANI>96% and dDDH>70%). Strains HZ1^T^ and HLT1-21 showed ANI values of 99.1% and a dDDH value of 92.2%, indicating that they represent the same genomic species. Strains MDB2-24^T^ and MDT3-44 exhibited ANI values of 98.5% and a dDDH value of 87.9%, likewise supporting assignment to a single genomic species [21]. In contrast, all remaining inter-strain comparisons fell below widely used species thresholds: the highest non-conspecific values were ANI 93.87% (MDB2-24^T^ vs. MDT2-2^T^) and dDDH 51.8% (MDT2-2^T^ vs. MDT3-44), supporting the presence of six distinct genomic species among the eight isolates (Table S5).

Consistent with the phylogenomic topology (Fig. 1), each of the following type strains represents a separate genomic species: MDT1-65^T^, MDT2-2^T^, TMN-37^T^ and TMS1-12-1^T^, whereas HZ1^T^ and MDB2-24^T^ each represent genomic species that additionally include HLT1-21 and MDT3-44, respectively. Comparisons against the selected closest type-strain genomes were also below the 70% dDDH boundary, further supporting species-level novelty (Fig. 2). For example, the highest dDDH values to described relatives were 48.4% (MDT2-2^T^ vs. *Arthrobacter sedimenti* MIC A30^T^), 48.2% (MDB2-24^T^ vs. *A. sedimenti* MIC A30^T^) and 24.4% (HZ1^T^/HLT1-21 vs. *Arthrobacter flavus* JCM 11496^T^), consistent with species-level distinctiveness [21]. Therefore, these eight strains represent six novel species within the genus *Arthrobacter*.

Genome-relatedness indices strongly support the conclusion that *Arthrobacter vasquezii* Valenzuela-Ibaceta *et al.* 2023 [22] is a later heterotypic synonym of *Arthrobacter parietis* Heyrman *et al.* 2005 [23]. In the phylogenomic tree, *A. vasquezii* EH-1B-1^T^ and *A. parietis* JCM 14917^T^ formed a single strongly supported lineage (Fig. 1). The genome pair yielded ANI=98.8% and dDDH=90.1%, which exceed widely accepted genomic species thresholds (95–96% ANI; 70% dDDH), indicating conspecificity. Therefore, *A*. *vasquezii* Valenzuela-Ibaceta *et al.* 2023 should be regarded as a later heterotypic synonym of *A. parietis* Heyrman *et al.* 2005, based on the principle of priority. This genomic conclusion is further consistent with the published phenotypic and chemotaxonomic descriptions. Strain *A. parietis* LMG 22281^T^ was described as Gram-positive, non-motile, showing a rod-coccus morphology, catalase-positive and oxidase-negative, with good growth at low temperature (growth after 1 week at 4 °C) and a DNA G+C content of 63.8 mol%. Its predominant fatty acids were anteiso-C_15 : 0_ and iso-C_15 : 0_ (51% and 29%) [23]. Strain *A. vasquezii* EH-1B-1^T^ is likewise described as non-motile with a rod-coccus cycle, catalase-positive, with a genome-based DNA G+C content of 63.1 mol%, and a fatty-acid profile dominated by anteiso-C_15 : 0_ and iso-C_15 : 0_ (45.3% and 22.9%, respectively) [22]. Minor discrepancies between published biochemical readouts (e.g. oxidase reaction) are plausibly attributable to methodological differences, incubation conditions and strain-to-strain variability and do not outweigh the genome-scale evidence for conspecificity.

## Physiology and chemotaxonomy

Phenotypic characterization was performed using cultures grown on PYG agar for 3 days unless stated otherwise. Colony morphology was recorded visually. Gram staining and hydrolysis of casein, starch and Tween 80 were examined as described by Smibert and Krieg [24]. Cell morphology was observed by transmission electron microscopy (JEM-1400; JEOL Ltd., Tokyo, Japan) after preparing suspensions of PYG-grown cells in deionized water, mounting onto copper grids and negative staining with phosphotungstic acid for 5–10 s. Catalase activity was determined by effervescence in 3% (v/v) H_2_O_2_, and oxidase activity was evaluated using 1% (w/v) tetramethyl-p-phenylenediamine. Growth was assessed in PYG broth at 0–37 °C, across initial pH 4.0–11.0, and with 0–7.0% (w/v) NaCl (0.5% increments). Growth at 0 °C was tested in liquid PYG medium by incubation in an ice–water bath maintained at ~0 °C for 15 days. Growth was evaluated by an increase in turbidity relative to uninoculated controls. pH growth tests were based on initially adjusted medium pH values; terminal pH values were not remeasured, and these data are, therefore, used only as descriptive characters rather than as primary species-circumscription evidence. Substrate utilization was tested using API 50 CH strips (bioMérieux, France) in a defined basal medium containing 0.2% (NH_4_)_2_SO_4_, 0.05% NaH_2_PO_4_·H_2_O, 0.05% K_2_HPO_4_, 0.02% MgSO_4_·7H_2_O and 0.01% CaCl_2_·2H_2_O. Additional physiological and enzymatic traits were evaluated using API 20NE, API 20E and API ZYM systems (bioMérieux) according to the supplier’s protocols. For cellular fatty acid profiling, biomass was harvested from PYG plates at late exponential phase after incubation at 14 °C. Fatty acid methyl esters were prepared following the MIDI protocol (Sherlock Microbial Identification System, MIDI 6.0) [25] and analysed by gas chromatography on an Agilent 6890 N instrument equipped with an HP-ULTRA 2 capillary column (25 m × 0.2 mm × 0.33 µm, Agilent). Fatty acids were identified using the TSBA6 library.

Six representative strains were selected for physiology and chemotaxonomy analyses. All six strains were catalase-positive and oxidase-negative, and no flagella were observed in the tested conditions (Table 1). Growth occurred at low temperature in all strains (minimum 0 °C in the tested range), but the upper temperature limits differed: HZ1^T^ grew up to 26 °C, whereas TMN-37^T^ and MDB2-24^T^ grew up to 36 °C. The relatively high maximum growth temperatures indicate that these strains have broad temperature ranges for growth. However, the upper growth-temperature limit alone should not be interpreted as evidence of ecological adaptation to glacier habitats. Cold adaptation is more appropriately evaluated by integrating multiple traits, including the capacity and rate of growth at low temperatures, tolerance to repeated freeze–thaw cycles and other physiological responses to cold stress. Salt tolerance also varied, with growth observed up to 5.5% NaCl for HZ1^T^ and TMS1-12-1^T^, up to 4.0% for TMN-37^T^ and up to 4.5% for MDT2-2^T^. A set of simple diagnostic reactions, including substrate hydrolysis and enzymatic activity assays, enabled the discrimination of the six proposed type strains. Carbon-source utilization patterns provided additional descriptive separation. Cellular fatty acid profiles were broadly consistent with assignment to *Arthrobacter* and provided supplementary diagnostic information. In all six type strains, anteiso-C_15 : 0_ was the predominant component (34.5–66.1%), accompanied by varying proportions of summed feature 3 (0–22.1%; C_16 : 1_* ω7*c and/or C_16 : 1_* ω6*c), anteiso-C_17 : 1_* ω9*c (0–17.1%), anteiso-C_17 : 1_ A (0–12.5%) and anteiso-C_17 : 0_ (2.8–9.0%) (Table S6). Strain TMN-37^T^ displayed an unusually high proportion of anteiso-C_15 : 0_ (66.1%), whereas HZ1^T^ contained comparatively elevated anteiso-C_17 : 1_* ω9*c (17.1%) and MDB2-24^T^ and TMS1-12-1^T^ showed higher iso-C_16 : 1_ h (11.7% and 13.0%, respectively) relative to several other strains.

**Table 1.** Differential phenotypic characteristics of the six type strains isolated in this study Strains: 1, HZ1^T^; 2, TMS1-12-1^T^; 3, TMN-37^T^; 4, MDT1-65^T^; 5, MDB2-24^T^; 6, MDT2-2^T^. +, Positive; −, negative; w, weak positive.

Characteristic	1	2	3	4	5	6
Growth temperature (°C)	0–26	0–34	0–36	0–33	0–36	0–33
Hydrolysis of casein	−	+	−	−	−	−
Hydrolysis of Tween 80	+	+	+	−	+	+
Hydrolysis of aesculin	+	−	+	+	+	+
Hydrolysis of gelatin	−	+	−	+	−	+
Voges–Proskauer test	+	−	−	+	+	+
Citrate utilization	+	−	−	−	−	−
**Utilization of**						
Glycerol	−	+	+	−	+	−
l-Arabinose	−	+	−	−	+	−
d-Ribose	−	−	+	−	+	−
d-Xylose	−	+	−	−	+	−
d-Galactose	−	+	+	−	+	−
d-Glucose	−	+	+	+	+	−
d-Fructose	−	+	+	−	+	−
d-Mannose	−	+	+	+	+	−
l-Rhamnose	−	−	−	−	+	−
d-Mannitol	−	+	+	+	+	−
d-Sorbitol	−	−	−	+	−	−
Amygdalin	−	−	−	−	+	−
Arbutin	−	+	−	−	+	−
Aesculin	−	+	−	−	+	+
Salicin	−	−	+	−	+	−
d-cellobiose	−	+	+	−	+	−
d-maltose	−	+	+	−	+	−
d-melibiose	−	−	−	+	−	−
d-sucrose	−	+	+	−	+	−
d-trehalose	−	+	−	+	w	−
d-melezitose	−	−	+	−	−	−
d-raffinose	−	−	+	+	−	−
Glycogen	+	+	−	−	−	−
Xylitol	−	−	−	+	−	−
d-turanose	−	+	+	+	+	−
l-Arabitol	−	−	−	+	−	−

In summary, phylogenomics and genome relatedness provide the primary basis for recognizing six separate species among the eight glacier-derived *Arthrobacter* strains. Phenotypic and chemotaxonomic data – including differences in growth temperature range, NaCl tolerance, hydrolytic and biochemical reactions, enzyme activities, carbon utilization patterns and fatty acid composition – provide descriptive and diagnostic support for the six species. In addition, based on genome relatedness and the principle of priority, *A. vasquezii* Valenzuela-Ibaceta *et al.* 2023 should be considered a later heterotypic synonym of *A. parietis* Heyrman *et al.* 2005. Therefore, we propose the following six species names within the genus *Arthrobacter*:

1. ***Arthrobacter glycogeni*** **sp. nov**. (strains HZ1^T^, HLT1-21; type strain HZ1^T^=CGMCC 1.9261^T^=JCM 38152^T^)
2. ***Arthrobacter arabinosi* sp. nov**. (type strain TMS1-12-1^T^=CGMCC 1.9392^T^=NBRC 113087^T^)
3. ***Arthrobacter melezitosi* sp. nov**. (type strain TMN-37^T^=CGMCC 1.9692^T^=JCM 38156^T^)
4. ***Arthrobacter sorbitolis* sp. nov**. (type strain MDT1-65^T^=CGMCC 1.9836^T^=JCM 38163^T^)
5. ***Arthrobacter rhamnosi* sp. nov**. (strains MDB2-24^T^, MDT3-44; type strain MDB2-24^T^=CGMCC 1.9862^T^=JCM 38160^T^)
6. ***Arthrobacter pasteuri* sp. nov**. (type strain MDT2-2^T^=CGMCC 1.9874^T^=JCM 38164^T^)

## Protologues

### Description of *Arthrobacter glycogeni* sp. nov.

*Arthrobacter glycogeni* (gly.co.ge’ni. N.L. gen. n. *glycogeni*, of glycogen).

Cells are Gram-stain-positive, aerobic, non-motile, rod-shaped, ~0.6–0.7 µm wide by 1.0–1.2 µm long. Colonies are lemon-yellow, convex, circular, smooth and opaque on PYG plates at 14 °C. Growth occurs at 0–26 °C (no growth at 28 °C) and at pH 7.0–11.0 (optimum pH 7.0) and NaCl tolerance up to 5.5% (w/v). Catalase-positive and oxidase-negative. Hydrolyses Tween 80 and aesculin, but does not hydrolyse starch, casein or gelatin. Indole and H_2_S are not produced. Glucose is not fermented. Nitrate is not reduced. Voges–Proskauer test is positive. Positive for citrate utilization, arginine dihydrolase, urease, *β*-galactosidase, esterase (C4), esterase lipase (C8) and leucine arylamidase. Negative for lysine decarboxylase, ornithine decarboxylase, tryptophan deaminase, alkaline phosphatase, lipase (C14), valine arylamidase, cystine arylamidase, trypsin, *α*-chymotrypsin, acid phosphatase, naphthol-AS-BI-phosphohydrolase, *α*-galactosidase, *β*-glucuronidase, *α*-glucosidase, *β*-glucosidase, *N*-acetyl-*β*-glucosaminidase, *α*-mannosidase and *α*-fucosidase. Utilize the following carbon sources: methyl-*α*-d-glucopyranoside, starch, glycogen, l-fucose and gluconate. Cannot utilize the following carbon sources: glycerol, erythritol, d-arabinose, l-arabinose, d-ribose, d-xylose, l-xylose, d-adonitol, methyl-*β*-d-xylopyranoside, d-galactose, d-glucose, d-fructose, d-mannose, l-sorbose, l-rhamnose, dulcitol, inositol, d-mannitol, d-sorbitol, methyl-*α*-d-mannopyranoside, *N*-acetylglucosamine, amygdalin, arbutin, aesculin, salicin, d-cellobiose, d-maltose, d-lactose, d-melibiose, d-sucrose, d-trehalose, inulin, d-melezitose, d-raffinose, xylitol, gentiobiose, d-turanose, d-lyxose, d-tagatose, d-fucose, d-arabitol, l-arabitol, 2-ketogluconate and 5-ketogluconate. The major fatty acids (>10%) include anteiso-C_15 : 0_, anteiso-C_17 : 1_* ω9*c and summed feature 3 (C_16 : 1_* ω7*c and/or C_16 : 1_* ω6*c). The DNA G+C content of the type strain is 63.8 mol%.

The type strain, HZ1^T^ (=CGMCC 1.9261^T^=JCM 38152^T^), was isolated from a cryoconite sample collected from Xinjiang No. 1 Glacier, Xinjiang Uygur Autonomous Region, P.R. China. The GenBank/EMBL/DDBJ accession numbers for the 16S rRNA gene and whole-genome sequences of HZ1^T^ are JX949320 and JBTNDE000000000, respectively.

### Description of *Arthrobacter arabinosi* sp. nov.

*Arthrobacter arabinosi* (a.ra.bi.no’si. N.L. gen. n. *arabinosi*, of arabinose).

Cells are Gram-stain-positive, aerobic, non-motile, short rods and cocci (diameter 0.5–0.7 µm). Colonies are pink, convex, circular, smooth and opaque on PYG plates at 14 °C. Growth occurs at 0–34 °C (no growth at 36 °C) and at pH 7.0–10.0 (optimum pH 7.0) and NaCl tolerance up to 5.5% (w/v). Catalase-positive and oxidase-negative. Hydrolyses starch, casein, Tween 80 and gelatin, but does not hydrolyse aesculin. Indole and H_2_S are not produced. Glucose is not fermented. Nitrate is not reduced. Voges–Proskauer test is negative. Positive for *β*-galactosidase, alkaline phosphatase and leucine arylamidase. Negative for citrate utilization, lysine decarboxylase, ornithine decarboxylase, tryptophan deaminase, arginine dihydrolase, urease, esterase (C4), esterase lipase (C8), lipase (C14), valine arylamidase, cystine arylamidase, trypsin, *α*-chymotrypsin, acid phosphatase, naphthol-AS-BI-phosphohydrolase, *α*-galactosidase, *β*-glucuronidase, *α*-glucosidase, *β*-glucosidase, *N*-acetyl-*β*-glucosaminidase, *α*-mannosidase and *α*-fucosidase. Utilize the following carbon sources: glycerol, l-arabinose, d-xylose, d-galactose, d-glucose, d-fructose, d-mannose, d-mannitol, arbutin, aesculin, d-cellobiose, d-maltose, d-lactose, d-sucrose, d-trehalose, starch, glycogen, gentiobiose, d-turanose and gluconate. Cannot utilize the following carbon sources: erythritol, d-arabinose, d-ribose, l-xylose, d-adonitol, methyl-*β*-d-xylopyranoside, l-sorbose, l-rhamnose, dulcitol, inositol, d-sorbitol, methyl-*α*-d-mannopyranoside, methyl-*α*-d-glucopyranoside, *N*-acetylglucosamine, amygdalin, salicin, d-melibiose, inulin, d-melezitose, d-raffinose, xylitol, d-lyxose, d-tagatose, d-fucose, l-fucose, d-arabitol, l-arabitol, 2-ketogluconate and 5-ketogluconate. The major fatty acids (>10%) include anteiso-C_15 : 0_, iso-C_16 : 1_ h, anteiso-C_17 : 1_* ω9*c and summed feature 3 (C_16 : 1_* ω7*c and/or C_16 : 1_* ω6*c). The DNA G+C content of the type strain is 69.4 mol%.

The type strain, TMS1-12-1^T^ (=CGMCC 1.9392^T^=NBRC 113087^T^), was isolated from an ice sample collected from the Toumingmengke Glacier, Gansu Province, P.R. China. The GenBank/EMBL/DDBJ accession numbers for the 16S rRNA gene and whole-genome sequences of TMS1-12-1^T^ are JX949834 and JBTNDF000000000, respectively.

### Description of *Arthrobacter melezitosi* sp. nov.

*Arthrobacter melezitosi* (me.le.zi’to.si. N.L. gen. n. *melezitosi*, of melezitose).

Cells are Gram-stain-positive, aerobic, non-motile, short rods and cocci (diameter 1.0–1.8 µm). Colonies are pale pink, convex, circular, smooth and opaque on PYG plates at 14 °C. Growth occurs at 0–36 °C (no growth at 40 °C) and at pH 6.0–7.0 (optimum pH 7.0; weak growth at pH 8.0) and NaCl tolerance up to 4.0% (w/v). Catalase-positive and oxidase-negative. Hydrolyses Tween 80, starch and aesculin, but does not hydrolyse casein or gelatin. Indole and H_2_S are not produced. Glucose is not fermented. Nitrate is not reduced. Voges–Proskauer test is negative. Positive for *β*-galactosidase, leucine arylamidase, valine arylamidase and cystine arylamidase. Negative for citrate utilization, lysine decarboxylase, ornithine decarboxylase, tryptophan deaminase, arginine dihydrolase, urease, alkaline phosphatase, esterase (C4), esterase lipase (C8), lipase (C14), trypsin, *α*-chymotrypsin, acid phosphatase, naphthol-AS-BI-phosphohydrolase, *α*-galactosidase, *β*-glucuronidase, *α*-glucosidase, *β*-glucosidase, *N*-acetyl-*β*-glucosaminidase, *α*-mannosidase and *α*-fucosidase. Utilize the following carbon sources: glycerol, d-ribose, d-galactose, d-glucose, d-fructose, d-mannose, d-mannitol, salicin, d-cellobiose, d-maltose, d-sucrose, d-melezitose, d-raffinose, d-turanose and gluconate. Cannot utilize the following carbon sources: erythritol, d-arabinose, l-arabinose, d-xylose, l-xylose, d-adonitol, methyl-*β*-d-xylopyranoside, l-sorbose, l-rhamnose, dulcitol, inositol, d-sorbitol, methyl-*α*-d-mannopyranoside, methyl-*α*-d-glucopyranoside, *N*-acetylglucosamine, amygdalin, arbutin, aesculin, d-lactose, D-melibiose, d-trehalose, inulin, starch, glycogen, xylitol, gentiobiose, d-lyxose, d-tagatose, d-fucose, l-fucose, d-arabitol, l-arabitol, 2-ketogluconate and 5-ketogluconate. The major fatty acid (>10%) is anteiso-C_15 : 0_. The DNA G+C content of the type strain is 68.8 mol%.

The type strain, TMN-37^T^ (=CGMCC 1.9692^T^=JCM 38156^T^), was isolated from an ice sample collected from the Toumingmengke Glacier, Gansu Province, P.R. China. The GenBank/EMBL/DDBJ accession numbers for the 16S rRNA gene and whole-genome sequences of TMN-37^T^ are JX949861 and JBTNDJ000000000, respectively.

### Description of *Arthrobacter sorbitolis* sp. nov.

*Arthrobacter sorbitolis* (sor.bi.to’lis. N.L. gen. n. *sorbitolis*, of sorbitol).

Cells are Gram-stain-positive, aerobic, non-motile, short rods and cocci, ~0.5–0.6 µm wide by 0.9–1.0 µm long. Colonies are pink, convex, circular, smooth and opaque on PYG plates at 14 °C. Growth occurs at 0–33 °C (no growth at 35 °C) and at pH 7.0–10.0 (optimum pH 7.0; weak growth at pH 6.0) and NaCl tolerance up to 5.5% (w/v) (weak growth at 6.0%). Catalase-positive and oxidase-negative. Hydrolyses gelatin and aesculin, but does not hydrolyse starch, casein or Tween 80. Indole and H_2_S are not produced. Glucose is not fermented. Nitrate is not reduced. Voges–Proskauer test is positive. Positive for *β*-galactosidase, alkaline phosphatase, esterase (C4), esterase lipase (C8), leucine arylamidase, naphthol-AS-BI-phosphohydrolase and *α*-glucosidase. Negative for citrate utilization, lysine decarboxylase, ornithine decarboxylase, tryptophan deaminase, arginine dihydrolase, urease, lipase (C14), valine arylamidase, cystine arylamidase, trypsin, *α*-chymotrypsin, acid phosphatase, *α*-galactosidase, *β*-glucuronidase, *β*-glucosidase, *N*-acetyl-*β*-glucosaminidase, *α*-mannosidase and *α*-fucosidase. Utilize the following carbon sources: d-glucose, d-mannose, d-mannitol, d-sorbitol, d-melibiose, d-trehalose, d-raffinose, xylitol, d-turanose, l-arabitol and gluconate. Cannot utilize the following carbon sources: glycerol, erythritol, d-arabinose, l-arabinose, d-ribose, d-xylose, l-xylose, d-adonitol, methyl-*β*-d-xylopyranoside, d-galactose, d-fructose, l-sorbose, l-rhamnose, dulcitol, inositol, methyl-*α*-d-mannopyranoside, methyl-*α*-d-glucopyranoside, *N*-acetylglucosamine, amygdalin, arbutin, aesculin, salicin, d-cellobiose, d-maltose, d-lactose, d-sucrose, inulin, d-melezitose, starch, glycogen, gentiobiose, d-lyxose, d-tagatose, d-fucose, l-fucose, d-arabitol, 2-ketogluconate and 5-ketogluconate. The major fatty acids (>10%) include anteiso-C_15 : 0_, anteiso-C_17 : 1_ A and summed feature 3 (C_16 : 1_* ω7*c and/or C_16 : 1_* ω6*c). The DNA G+C content of the type strain is 69.6 mol%.

The type strain, MDT1-65^T^ (=CGMCC 1.9836^T^=JCM 38163^T^), was isolated from an ice sample collected from the Midui Glacier, Tibet Autonomous Region, P.R. China. The GenBank/EMBL/DDBJ accession numbers for the 16S rRNA gene and whole-genome sequences of MDT1-65^T^ are JX949665 and JBTNDM000000000, respectively.

### Description of *Arthrobacter rhamnosi* sp. nov.

*Arthrobacter rhamnosi* (rham.no’si. N.L. gen. n. *rhamnosi*, of rhamnose).

Cells are Gram-stain-positive, aerobic, non-motile, short rods and cocci (diameter 0.9–1.3 µm). Colonies are pink, convex, circular, smooth and opaque on PYG plates at 14 °C. Growth occurs at 0–36 °C (no growth at 40 °C) and at pH 6.0–10.0 (optimum pH 7.0; weak growth at pH 11.0) and NaCl tolerance up to 5.0% (w/v). Catalase-positive and oxidase-negative. Hydrolyses starch, Tween 80 and aesculin, but does not hydrolyse casein or gelatin. Indole and H_2_S are not produced. Glucose is not fermented. Nitrate is not reduced. Voges–Proskauer test is positive. Positive for *β*-galactosidase, alkaline phosphatase, leucine arylamidase, naphthol-AS-BI-phosphohydrolase and *α*-glucosidase. Negative for citrate utilization, lysine decarboxylase, ornithine decarboxylase, tryptophan deaminase, arginine dihydrolase, urease, esterase (C4), esterase lipase (C8), lipase (C14), valine arylamidase, cystine arylamidase, trypsin, *α*-chymotrypsin, acid phosphatase, *α*-galactosidase, *β*-glucuronidase, *β*-glucosidase, *N*-acetyl-*β*-glucosaminidase, *α*-mannosidase and *α*-fucosidase. Utilize the following carbon sources: glycerol, l-arabinose, d-ribose, d-xylose, methyl-*β*-d-xylopyranoside, d-galactose, d-glucose, d-fructose, d-mannose, l-rhamnose, d-mannitol, methyl-α-d-glucopyranoside, *N*-acetylglucosamine, amygdalin, arbutin, aesculin, salicin, d-cellobiose, d-maltose, d-sucrose, d-trehalose, D-turanose and gluconate. Cannot utilize the following carbon sources: erythritol, d-arabinose, l-xylose, d-adonitol, l-sorbose, dulcitol, inositol, d-sorbitol, methyl-α-d-mannopyranoside, lactose, melibiose, inulin, melezitose, raffinose, starch, glycogen, xylitol, gentiobiose, d-lyxose, d-tagatose, d-fucose, l-fucose, d-arabitol, l-arabitol, 2-ketogluconate and 5-ketogluconate. The major fatty acids (>10%) include anteiso-C_15 : 0_, iso-C_16 : 1_ h, anteiso-C_17 : 1_ A and summed feature 3 (C_16 : 1_* ω*7*c* and/or C_16 : 1_* ω6*c). The DNA G+C content of the type strain is 68.5 mol%.

The type strain, MDB2-24^T^ (=CGMCC 1.9862^T^=JCM 38160^T^), was isolated from an ice sample collected from the Midui Glacier, Tibet Autonomous Region, P.R. China. The GenBank/EMBL/DDBJ accession numbers for the 16S rRNA gene and whole-genome sequences of MDB2-24^T^ are JX949711 and JBTNDN000000000, respectively.

### Description of *Arthrobacter pasteuri* sp. nov.

*Arthrobacter pasteuri* (pas.teu’ri. N.L. gen. masc. n. *pasteuri*, of Pasteur; honouring the French microbiologist Louis Pasteur).

Cells are Gram-stain-positive, aerobic, non-motile, short rods and cocci (diameter 1.0–1.5 µm). Colonies are pink, convex, circular, smooth and opaque on PYG plates at 14 °C. Growth occurs at 0–33 °C (no growth at 35 °C) and at pH 7.0–10.0 (optimum pH 7.0; weak growth at pH 11.0) and NaCl tolerance up to 4.5% (w/v) (weak growth at 5.0%). Catalase-positive and oxidase-negative. Hydrolyses starch, Tween 80, gelatin and aesculin, but does not hydrolyse casein. Indole and H_2_S are not produced. Glucose is not fermented. Nitrate is not reduced. Voges–Proskauer test is positive. Positive for urease, *β*-galactosidase, alkaline phosphatase, esterase (C4), esterase lipase (C8), leucine arylamidase and *α*-glucosidase. Negative for citrate utilization, lysine decarboxylase, ornithine decarboxylase, tryptophan deaminase, arginine dihydrolase, lipase (C14), valine arylamidase, cystine arylamidase, trypsin, *α*-chymotrypsin, acid phosphatase, naphthol-AS-BI-phosphohydrolase, *α*-galactosidase, *β*-glucuronidase, *β*-glucosidase, *N*-acetyl-*β*-glucosaminidase, *α*-mannosidase and *α*-fucosidase. Utilize the following carbon sources: aesculin and gluconate. Cannot utilize the following carbon sources: glycerol, erythritol, d-arabinose, l-arabinose, d-ribose, d-xylose, l-xylose, d-adonitol, methyl-*β*-d-xylopyranoside, d-galactose, d-glucose, d-fructose, d-mannose, l-sorbose, l-rhamnose, dulcitol, inositol, d-mannitol, d-sorbitol, methyl-*α*-d-mannopyranoside, methyl-*α*-d-glucopyranoside, *N*-acetylglucosamine, amygdalin, arbutin, salicin, d-cellobiose, d-maltose, d-lactose, d-melibiose, d-sucrose, d-trehalose, inulin, d-melezitose, d-raffinose, starch, glycogen, xylitol, gentiobiose, d-turanose, d-lyxose, d-tagatose, d-fucose, l-fucose, d-arabitol, l-arabitol, 2-ketogluconate and 5-ketogluconate. The major fatty acids (>10%) include anteiso-C_15 : 0_, anteiso-C_17 : 1_ A and summed feature 3 (C_16 : 1_* ω7*c and/or C_16 : 1_* ω6*c). The DNA G+C content of the type strain is 68.4 mol%.

The type strain, MDT2-2^T^ (=CGMCC 1.9874^T^=JCM 38164^T^), was isolated from a cryoconite sample collected from the Midui Glacier, Tibet Autonomous Region, P.R. China. The GenBank/EMBL/DDBJ accession numbers for the 16S rRNA gene and whole-genome sequences of MDT2-2^T^ are JX949668 and JBTNDO000000000, respectively.

## Supplementary material

10.1099/ijsem.0.007246 Supplementary Material 1.

## ABBREVIATIONS

AAI: average amino acid identity
ANI: average nucleotide identity
dDDH: digital DNA–DNA hybridization
ML: maximum-likelihood
NJ: neighbour-joining
PYG: peptone–yeast extract–glucose

## References

[1] Conn HJ, Dimmick I (1947). Soil Bacteria Similar in Morphology to *Mycobacterium* and *Corynebacterium*. J Bacteriol.

[2] Busse HJ (2016). Review of the taxonomy of the genus *Arthrobacter*, emendation of the genus *Arthrobacter sensu lato*, proposal to reclassify selected species of the genus *Arthrobacter* in the novel genera *Glutamicibacter* gen. nov., *Paeniglutamicibacter* gen. nov., *Pseudoglutamicibacter* gen. nov., *Paenarthrobacter* gen. nov. and *Pseudarthrobacter* gen. nov., and emended description of *Arthrobacter roseus*. Int J Syst Evol Microbiol.

[3] Liu Q, Xin YH, Zhou YG, Chen WX (2018). Multilocus sequence analysis of homologous recombination and diversity in *Arthrobacter sensu* lato named species and glacier-inhabiting strains. Syst Appl Microbiol.

[4] Pittino F, Maglio M, Gandolfi I, Azzoni RS, Diolaiuti G (2018). Bacterial communities of cryoconite holes of a temperate alpine glacier show both seasonal trends and year-to-year variability. Ann Glaciol.

[5] Liu Q, Zhou YG, Xin YH (2015). High diversity and distinctive community structure of bacteria on glaciers in China revealed by 454 pyrosequencing. Syst Appl Microbiol.

[6] Margesin R, Schumann P, Zhang D-C, Redzic M, Zhou Y-G (2012). *Arthrobacter cryoconiti* sp. nov., a psychrophilic bacterium isolated from alpine glacier cryoconite. Int J Syst Evol Microbiol.

[7] Yang LL, Liu HC, Liu Q, Xin YH (2021). *Arthrobacter cheniae* and *Arthrobacter frigidicola* sp. nov., isolated from a glacier. Int J Syst Evol Microbiol.

[8] Lane DJ, Stackebrandt E, Goodfellow M (1991). Nucleic Acid Techniques in Bacterial Systematics.

[9] Yoon S-H, Ha S-M, Kwon S, Lim J, Kim Y (2017). Introducing EzBioCloud: a taxonomically united database of 16S rRNA gene sequences and whole-genome assemblies. Int J Syst Evol Microbiol.

[10] Katoh K, Standley DM (2013). MAFFT multiple sequence alignment software version 7: improvements in performance and usability. *Mol Biol Evol*.

[11] Kumar S, Stecher G, Suleski M, Sanderford M, Sharma S (2024). MEGA12: molecular evolutionary genetics analysis version 12. Mol Biol Evol.

[12] Bankevich A, Nurk S, Antipov D, Gurevich AA, Dvorkin M (2012). SPAdes: a new genome assembly algorithm and its applications to single-cell sequencing. J Comput Biol.

[13] Gurevich A, Saveliev V, Vyahhi N, Tesler G (2013). QUAST: quality assessment tool for genome assemblies. Bioinformatics.

[14] Chklovski A, Parks DH, Woodcroft BJ, Tyson GW (2023). CheckM2: a rapid, scalable and accurate tool for assessing microbial genome quality using machine learning. Nat Methods.

[15] Seemann T (2014). Prokka: rapid prokaryotic genome annotation. Bioinformatics.

[16] Kim J, Na S-I, Kim D, Chun J (2021). UBCG2: Up-to-date bacterial core genes and pipeline for phylogenomic analysis. J Microbiol.

[17] Castresana J (2000). Selection of conserved blocks from multiple alignments for their use in phylogenetic analysis. Mol Biol Evol.

[18] Minh BQ, Schmidt HA, Chernomor O, Schrempf D, Woodhams MD (2020). IQ-TREE 2: new models and efficient methods for phylogenetic inference in the genomic era. Mol Biol Evol.

[19] Jain C, Rodriguez-R LM, Phillippy AM, Konstantinidis KT, Aluru S (2018). High throughput ANI analysis of 90K prokaryotic genomes reveals clear species boundaries. Nat Commun.

[20] Meier-Kolthoff JP, Göker M (2019). TYGS is an automated high-throughput platform for state-of-the-art genome-based taxonomy. Nat Commun.

[21] Riesco R, Trujillo ME (2024). Update on the proposed minimal standards for the use of genome data for the taxonomy of prokaryotes. Int J Syst Evol Microbiol.

[22] Valenzuela-Ibaceta F, Carrasco V, Lagos-Moraga S, Dietz-Vargas C, Navarro CA (2023). *Arthrobacter vasquezii* sp. nov., isolated from a soil sample from Union Glacier, Antarctica. Int J Syst Evol Microbiol.

[23] Heyrman J, Verbeeren J, Schumann P, Swings J, De Vos P (2005). Six novel Arthrobacter species isolated from deteriorated mural paintings. Int J Syst Evol Microbiol.

[24] Smibert RM, Krieg NR, Gerhardt P, Murray RGE, Wood WA, Krieg NR (1994). Methods for General and Molecular Bacteriology.

[25] Sasser M (1990). Technical Note 101: Identification of Bacteria by Gas Chromatography of Cellular Fatty Acids.

