# Supplementary material for: Polyphasic taxonomy of glacier-derived Arthrobacter strains reveals six novel species within the Arthrobacter agilis group and indicates Arthrobacter vasquezii Valenzuela-Ibaceta et al. 2023 as a later heterotypic synonym of Arthrobacter parietis Heyrman et al. 2005
Source: Int J Syst Evol Microbiol. 2026 Jul 21;76(7):007246. doi: 10.1099/ijsem.0.007246 (PMC13387620; doi:10.1099/ijsem.0.007246)
Supplement: Supplementary Material 1. [file ijsem-76-07246-s001.pdf]

Polyphasic taxonomy of glacier-derived *Arthrobacter* strains reveals six novel species within the *Arthrobacter agilis* group and indicates *Arthrobacter vasquezii* Valenzuela-Ibaceta *et al*. 2023 as a later heterotypic synonym of *Arthrobacter parietis* Heyrman *et al*. 2005

Lei-Lei Yang, Yu-Hua Xin, Qing Liu

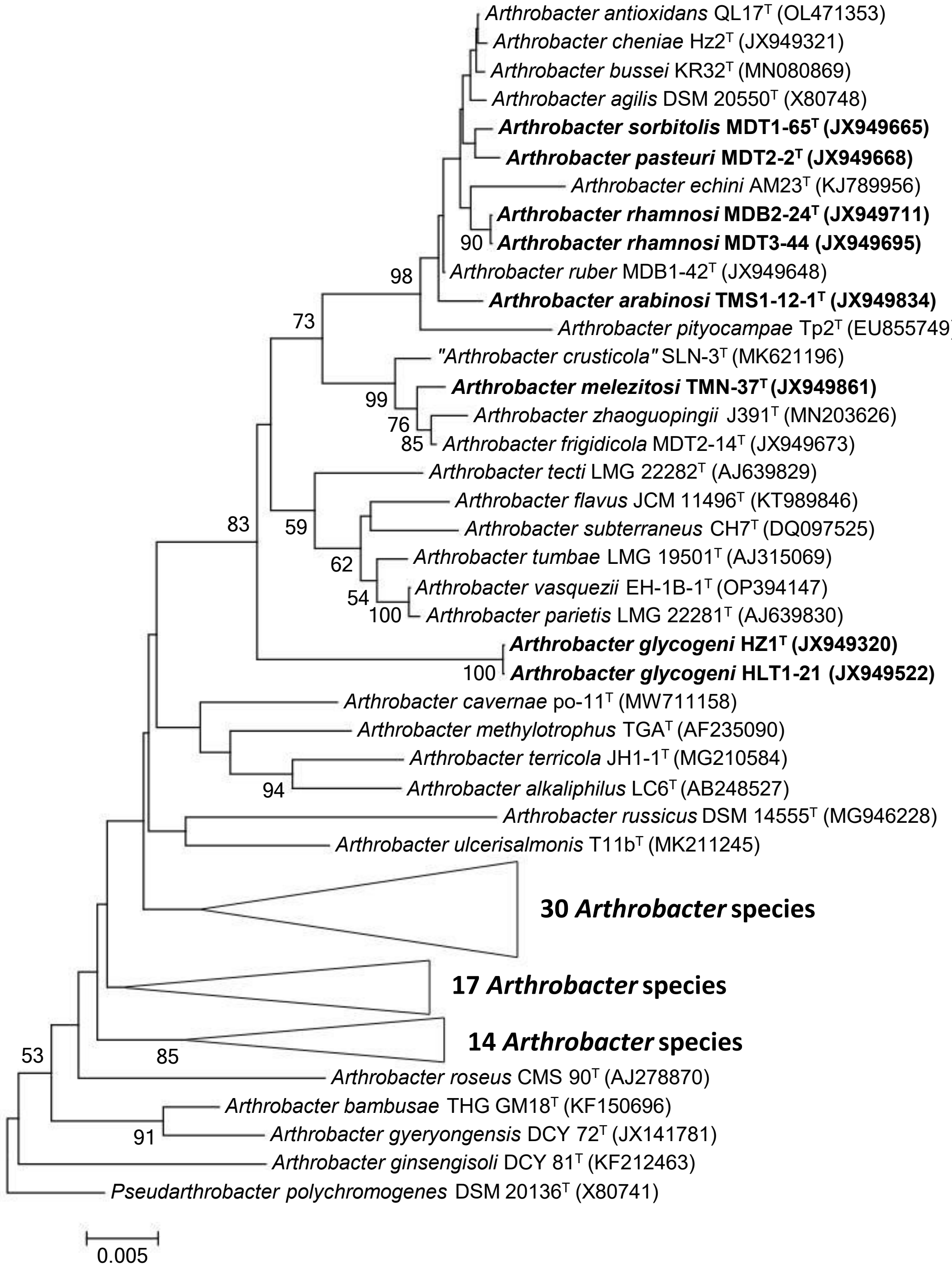

Fig. S1. Phylogenetic tree of the eight strains isolated in this study and related taxa based on 16S rRNA gene sequence comparisons using the NJ method. GenBank accession numbers are provided in parentheses. Bootstrap values (>50%) from 1,000 replicates are shown at branch nodes. Scale bar, 0.005 substitutions per nucleotide position.

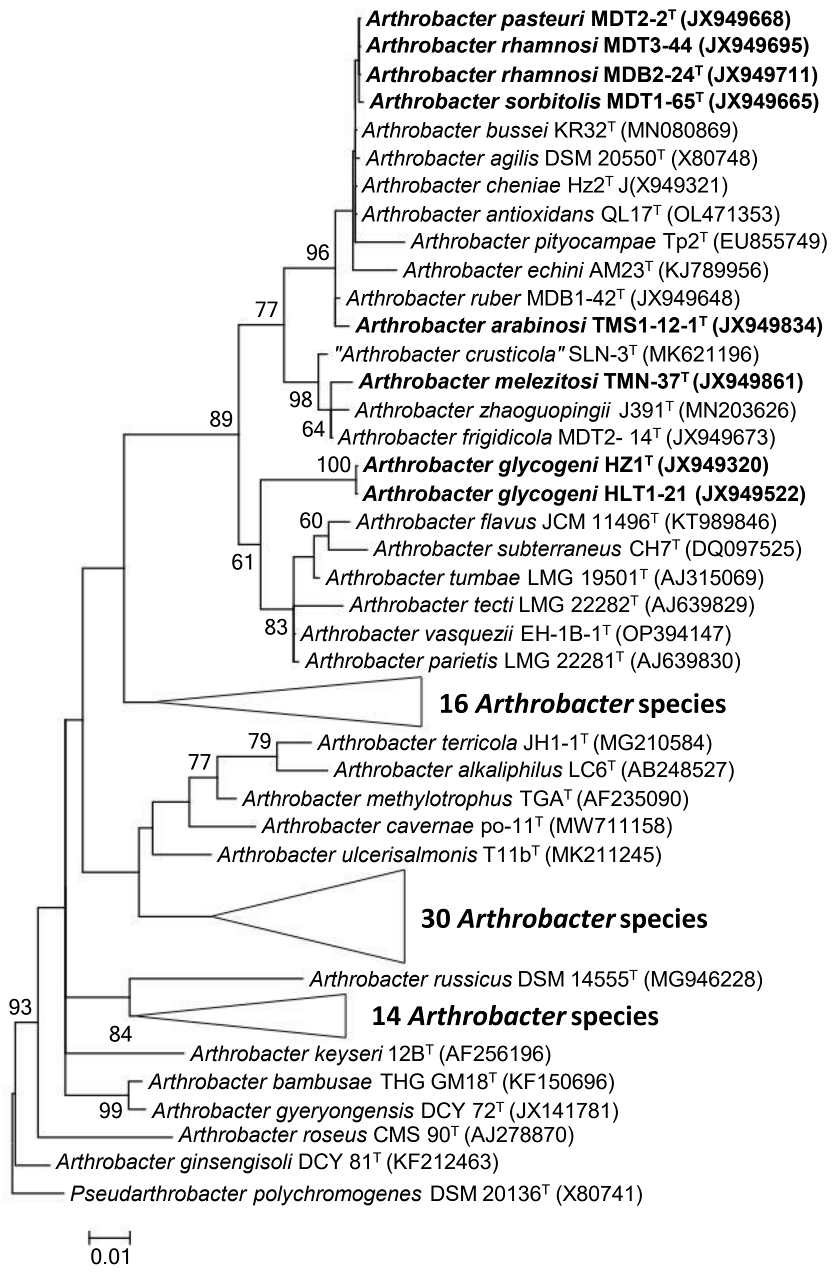

Fig. S2. Phylogenetic tree of the eight strains isolated in this study and related taxa based on 16S rRNA gene sequence comparisons using the ML method. GenBank accession numbers are provided in parentheses. Bootstrap values (>50%) from 1,000 replicates are shown at branch nodes. Scale bar, 0.01 substitutions per nucleotide position.

Table S1. The isolation information of the eight strains

Strain	Isolation location	Isolation source	Elevation (m a.s.l.)	Longitude (°E)	Latitude (°N)
**HZ1^T**	Xinjiang No. 1 Glacier	cryoconite	3,838	86.81	43.12
**TMS1-12-1^T**	Gansu Toumingmengke Glacier	cryoconite	4,278	39.496183	96.52295
**TMN-37^T**	Gansu Toumingmengke Glacier	cryoconite	4,278	39.496183	96.52295
**MDT1-65^T**	Xizang Midui Glacier	cryoconite	3,901	96.505000	29.451944
**MDB2-24^T**	Xizang Midui Glacier	ice	3,901	96.505000	29.451944
**MDT2-2^T**	Xizang Midui Glacier	cryoconite	3,901	96.505000	29.451944
**MDT3-44**	Xizang Midui Glacier	cryoconite	3,901	96.505000	29.451944
**HLT1-21**	Sichuan Hailuogou Glacier	cryoconite	3,457	101.968611	29.555833

Table S2. Reference genomes of type strains from related *Arthrobacter* taxa used for phylogenomic and genome-relatedness analyses, including NCBI RefSeq/GenBank accession numbers. All reference genomes listed in this table were included in the ANI analysis. The closest type-strain relatives identified from the ANI results and phylogenomic analysis were subsequently selected for dDDH comparison, as shown in Fig. 2.

Strain	Genome Accession Number
Arthrobacter agilis CCM 2390^T	GCA_042646785.1
Arthrobacter alkaliphilus JCM 21827^T	GCA_054511375.1
Arthrobacter alpinus DSM 22274^T	GCA_900105965.1
Arthrobacter antibioticus H35-MC1^T	GCA_030063205.1
Arthrobacter antioxidans QL17^T	GCA_023100725.1
Arthrobacter bambusae JCM 19335^T	GCA_054783335.1
Arthrobacter bussei KR32^T	GCA_009377195.2
Arthrobacter caoxuetaonis zg-Y453^T	GCA_023921125.1
Arthrobacter castelli DSM 16402^T	GCA_000430705.1
Arthrobacter cavernae PO-11^T	GCA_017368795.1
Arthrobacter celericrescens NEAU-SA2^T	GCA_003614925.1
Arthrobacter cheniae Hz2^T	GCA_003602275.1
Arthrobacter citreus DSM 20133^T	GCA_009192745.1
"*Arthrobacter crusticola*" SLN-3^T	GCA_004357995.1
Arthrobacter cryoconiti Cr6-08^T	GCA_020905375.1
Arthrobacter crystallopoietes DSM 20117^T	GCA_002849715.1
Arthrobacter cupressi DSM 24664^T	GCA_013409905.1
Arthrobacter deserti CGMCC 1.15091^T	GCA_012395865.1
Arthrobacter dokdonensis DCT-5^T	GCA_003268655.1
Arthrobacter echini AM23^T	GCA_004803505.1
Arthrobacter endolithicus H14-L1^T	GCA_026627425.1
Arthrobacter flavus JCM 11496^T	GCA_039526005.1
Arthrobacter gallicola Sa2CUA1^T	GCA_014836775.1
Arthrobacter gandavensis JCM 13316^T	GCA_009192735.1
Arthrobacter gengyunqii zg-Y809^T	GCA_023022985.1
Arthrobacter ginkgonis JCM 30742^T	GCA_039540195.1
Arthrobacter glacialis HLT2-12-2^T	GCA_002909445.1
Arthrobacter globiformis NBRC 12137^T	GCA_000238915.2
Arthrobacter gyeryongensis JCM 18514^T	GCA_039543785.1
Arthrobacter halodurans DSM 21081^T	GCA_041877255.1
"*Arthrobacter hankyongi*" I2-34^T	GCA_022012395.1
Arthrobacter horti YJM1^T	GCA_030731045.1
Arthrobacter humicola JCM 15921^T	GCA_039531445.1
Arthrobacter ipis IA7^T	GCA_014694315.1
Arthrobacter jiangjiafuii zg-ZUI227^T	GCA_018622995.1
Arthrobacter jinronghuae zg-Y859^T	GCA_025244825.1
Arthrobacter koreensis DSM 16760^T	GCA_009193255.1
Arthrobacter liuii CGMCC 1.12778^T	GCA_014639275.1
Arthrobacter livingstonensis LI2^T	GCA_003219815.1
Arthrobacter luteolus DSM 13067^T	GCA_008973725.1
Arthrobacter mangrovi NBRC 112813^T	GCA_026011795.1
Arthrobacter methylotrophus JCM 13519^T	GCA_039539965.1
Arthrobacter mobilis E918^T	GCA_012395835.1
Arthrobacter monumenti DSM 16405^T	GCA_054511255.1
Arthrobacter nanjingensis A33^T	GCA_038405295.1
"*Arthrobacter nitrophenolicus*" SJCon^T	GCA_040546185.1
Arthrobacter oryzae DSM 25586^T	GCA_003634095.1
Arthrobacter parietis JCM 14917^T	GCA_039530425.1
Arthrobacter pascens DSM 20545^T	GCA_017052465.1
Arthrobacter phoenicis 1P04PC^T	GCA_047238215.1
Arthrobacter pigmenti DSM 16403^T	GCA_011927905.1

Arthrobacter pityocampae Tp2^T	GCA_002927275.1
Arthrobacter polaris C1-1^T	GCA_021398215.1
Arthrobacter psychrochitiniphilus DSM 23143^T	GCA_013408645.1
Arthrobacter psychrolactophilus B7^T	GCA_003219795.1
Arthrobacter pullicola Sa2BUA2^T	GCA_014836875.1
Arthrobacter ramosus JCM 1334^T	GCA_039535095.1
Arthrobacter rhizosphaerae CCNWLXL 1-35^T	GCA_023062595.1
Arthrobacter rhombi JCM 11678^T	GCA_039526245.1
Arthrobacter roseus DSM 14508^T	GCA_016907875.1
Arthrobacter ruber MDB1-42^T	GCA_002954225.1
Arthrobacter russicus DSM 14555^T	GCA_031454135.1
"*Arthrobacter sedimenti*" MIC A30^T	GCA_011750795.2
Arthrobacter silvisoli NEAU-SA1^T	GCA_003369445.1
Arthrobacter silviterrae DSM 27180^T	GCA_030813935.1
Arthrobacter stackebrandtii DSM 16005^T	GCA_017876675.1
Arthrobacter sunyaminii zg-ZUI122^T	GCA_018866305.1
Arthrobacter tecti JCM 21772^T	GCA_054511355.1
"*Arthrobacter terrae*" Z1-20^T	GCA_015708085.1
Arthrobacter terricola JH1-1^T	GCA_004354015.1
Arthrobacter tumbae DSM 16406^T	GCA_016907495.1
Arthrobacter ulcerisalmonis aT11b^T	GCA_900609065.1
Arthrobacter vasquezii EH-1B-1^T	GCA_029455255.1
"*Arthrobacter wenxiniae*" AETb3-4^T	GCA_013376105.1
Arthrobacter woluwensis DSM 10495^T	GCA_900105345.1
Arthrobacter yangruifuii 785^T	GCA_009192775.1
Arthrobacter zhangbolii zg-Y462^T	GCA_022869865.1
Arthrobacter zhaoguopingii J391^T	GCA_009828595.1
Arthrobacter zhaoxinii zg-Y815^T	GCA_025244925.1

Table S3. Basic genome information for the eight strains isolated in this study

Strain	Completeness (%)	Contamination (%)	Contigs	Total length (Mb)	GC (%)	N50	N75
**HZ1^T**	99.99	0.42	53	3.48	63.83	135230	72703
**TMS1-12-1^T**	100	0.37	38	3.59	69.38	716045	335964
**TMN-37^T**	99.91	0.1	34	3.44	68.83	233828	104290
**MDT1-65^T**	100	0.02	24	3.80	69.65	355705	137863
**MDB2-24^T**	100	0.09	15	3.60	68.51	476604	306329
**MDT2-2^T**	100	0.02	10	3.51	68.42	2317634	465866
**MDT3-44**	100	0.07	22	3.70	68.35	474858	251873
**HLT1-21**	100	0.18	32	3.55	63.74	196108	135967

Table S4. Genomic annotation for the eight strains

Strain	CDS	contigs	gene	rRNA	tRNA	tmRNA
**HZ1^T**	3293	3370	25	3	48	1
**TMS1-12-1^T**	3306	3382	20	5	50	1
**TMN-37^T**	3190	3260	14	3	52	1
**MDT1-65^T**	3508	3583	19	3	52	1
**MDB2-24^T**	3281	3361	25	3	51	1
**MDT2-2^T**	3223	3302	24	3	51	1
**MDT3-44**	3383	3467	28	3	52	1
**HLT1-21**	3390	3467	25	3	48	1

Table S5. Pairwise dDDH and ANI values (%) between the eight strains

**ANI/dDDH (%)**	**HZ1^T**	**TMS1-12-1^T**	**TMN-37^T**	**MDT1-65^T**	**MDB2-24^T**	**MDT2-2^T**	**MDT3-44**	**HLT1-21**
**HZ1^T**	—	19.6	19.5	19.5	19.5	19.8	19.6	**92.2**
**TMS1-12-1^T**	76.9	—	21.4	27.0	26.5	26.5	26.5	19.6
**TMN-37^T**	77.2	79.6	—	21.4	21.3	21.4	21.3	19.5
**MDT1-65^T**	76.9	84.5	79.5	—	27.1	27.0	27.1	19.5
**MDB2-24^T**	76.8	83.9	79.6	84.4	—	51.7	**87.9**	19.5
**MDT2-2^T**	76.9	83.8	79.5	84.4	93.9	—	51.8	19.7
**MDT3-44**	76.8	83.9	79.4	84.4	**98.5**	93.8	—	19.6
**HLT1-21**	**99.1**	76.9	77.2	76.9	76.8	76.9	76.7	—

Table S6. Cellular fatty acid composition of the six proposed type strains

Strains: 1, HZ1^T; 2, TMS1-12-1^T; 3, TMN-37^T; 4, MDT1-65^T; 5, MDB2-24^T; 6, MDT2-2^T. Major fatty acids (>10 %) are represented in bold. TR, traces (<1 %); –, not detected. *Summed features represent fatty acids that cannot be resolved reliably from another fatty acid under the chosen chromatographic conditions. The MIDI system groups these fatty acids together as one feature with a single percentage of the total. Summed features 3, $C_{16:1}$ $\omega 7c$/$C_{16:1}$ $\omega 6c$.

**Fatty acids**	1	2	3	4	5	6
**Saturated**						
iso-$C_{14:0}$	TR	1.9	–	–	1.7	TR
anteiso-$C_{15:0}$	**42.1**	**34.5**	**66.1**	**39.7**	**39.3**	**41.7**
iso-$C_{15:0}$	3.1	8.9	3.2	6.8	4.1	5.1
iso-$C_{16:0}$	2.1	5.6	4.8	2.6	7.2	6.6
$C_{16:0}$	2.8	1.0	TR	3.3	1.5	2.4
anteiso-$C_{17:0}$	9.0	2.8	8.9	6.2	3.3	6.5
**Unsaturated**						
anteiso-$C_{15:1}$ A	TR	1.6	3.5	TR	TR	0.0
iso-$C_{16:1}$ H	2.1	**13.0**	1.6	2.5	**11.7**	5.4
anteiso-$C_{17:1}$ A	–	–	–	**10.8**	**13.2**	**12.5**
anteiso-$C_{17:1}$ ω9c	**17.1**	**12.7**	7.6	–	–	–
$C_{18:1}$ ω9c	1.2	TR	TR	1.3	TR	TR
**Summed feature 3**	**16.8**	**11.5**	TR	**22.1**	**13.1**	**15.3**
